# Mobile health (mHealth) interventions for health promotion during the perinatal period in India: a scoping review

**DOI:** 10.3389/fgwh.2024.1427285

**Published:** 2024-11-27

**Authors:** Zara Small, Sophie Elizabeth Thompson, Ankita Sharma, Sreya Majumdar, Sudhir Raj Thout, Devarsetty Praveen, Jane Elizabeth Hirst

**Affiliations:** ^1^ Medical Sciences Division, University of Oxford, Oxford, United Kingdom; ^2^ Nuffield Department of Women’s & Reproductive Health, Medical Sciences Division, University of Oxford, Oxford, United Kingdom; ^3^ Women's Health Program, The George Institute for Global Health, Hyderabad, India; ^4^ School of Public Health, Prasanna School of Public Health, Manipal Academy of Higher Education, Manipal, India; ^5^ Better Care, The George Institute for Global Health, University of New South Wales, Newtown, NWS, Australia; ^6^ School of Public Health, The George Institute for Global Health, School of Public Health, Imperial College London, London, United Kingdom

**Keywords:** mHealth, perinatal, India, maternal, mobile, digital, health

## Abstract

**Introduction:**

Perinatal and maternal mortality rates remain high in India compared to global levels, and there is significant heterogeneity in outcomes across Indian states. Many mobile health (mHealth) interventions have been developed to improve maternal and infant health outcomes in India, however it is unclear how mHealth can best support women in this culturally and resource diverse setting. Therefore, we aimed to identify mHealth interventions targeting women and their families in the perinatal period in India, identify barriers and facilitators to their uptake, and future research directions.

**Methods:**

The Preferred Reporting Items for Systematic Reviews and Meta-Analyses and Joanna Briggs Institute guidelines for scoping reviews was used for study selection and screening and the mHealth evidence reporting and assessment checklist was used for evaluating mHealth interventions. PubMed, CINAHL, Global Health, and ACM digital library were searched for records up to 2 April 2023. Studies were included where women who were pregnant, planning for a child, or in the 12 months after delivery, and their families, living in India received health advice via a technological medium.

**Results:**

1,783 records were screened, 29 met the inclusion criteria, describing 22 different mHealth interventions. Most frequent behavioural targets for interventions were breastfeeding, antenatal nutrition, and infant healthcare. Most interventions communicated to women through one-way communication methods, most frequently SMS. Participants reported positive views of mHealth, reported facilitators included group communication, use of non-maternal informative content, and a pictorial information format. Reported barriers included household responsibilities, technical difficulties, difficulty accessing a phone and difficulty understanding, or misinterpreting messages.

**Discussion:**

We conclude that mHealth interventions are acceptable to women in India during the perinatal period. However, current interventions lack evidence of long term behavioural change and fail to report on features important in sustainability and scalability, namely network infrastructure, data security and interoperability. We propose the need for a framework to understand existing cultural beliefs and support structures to avoid early intervention failure. Future research should investigate multimodal mHealth interventions for behavioural change, identify the appropriate frequency and format of mHealth messages, and address access limitations such as shared mobile phone ownership, and illiteracy rates.

## 1 Introduction

Perinatal and maternal mortality rates are declining in India, with the country estimated to be on track to reach their maternal United Nations Sustainable Development Goals by 2030 (1). However, maternal and infant mortality remains high in India compared to global levels; in 2020 India was one of five countries that contributed to nearly half (49%) of global under-5 mortalities, and reported approximately 24,000 maternal deaths, accounting for 8.3% of global maternal deaths (2, 3). There is significant heterogeneity in maternal and neonatal health outcomes across Indian states. For instance, 92 percent of women in Andaman Nicobar make at least four antenatal care visits compared to 14 percent of women in Bihar (4, 5).

Reported barriers to the utilisation of obstetric care services in India include a lack of knowledge of perinatal care practices, limited family support to access obstetric care services, and long travel distances to healthcare facilities (6). With widespread and ever-increasing rates of mobile phone ownership across India many mobile health (mHealth) initiatives have been developed to encourage health promotion particularly in rural and resource limited areas (7, 8).

The World Health Organization (WHO) defines mHealth as ‘the use of mobile wireless technologies for health’, encompassing mobile phones, patient monitoring devices, personal digital assistants, and other wireless devices (8). Previously reported systematic reviews have shown that mHealth interventions can significantly improve health access and health seeking behaviours across low- and middle-income countries (7, 9). However, these reviews are limited by heterogeneity of data, and rarely evaluate the sustainability, or country-specific socio-cultural factors that may impact an intervention.

The purpose of this scoping review is to outline which mHealth interventions have been developed and used to encourage health promotion for women who are currently planning for a child, pregnant, or in the first twelve months post-partum in India. We aim to identify the scope of current interventions, including their locations, recipient characteristics and health behaviours targeted, types of technology used, as well as highlighting barriers and facilitators to intervention uptake and lessons for scale of interventions.

## 2 Methods

This review was developed in line with the Preferred Reporting Items for Systematic Reviews and Meta Analyses and Joanna Briggs Institute (JBI) guidelines for scoping reviews (10, 11). A review protocol was not published for this study. The PCC framework (population, concept, and context) framework was used to guide development of inclusion criteria, literature search, and structure of this review, as recommended by JBI methodology (12).

### 2.1 Population

We include women who are currently pregnant, planning for a child, or in the 12 months after delivery. We define planning for pregnancy as women of childbearing age to account for the high unplanned pregnancy rate in India (13). We have extended the population from women to their families to account for women who share a phone with family members.

### 2.2 Concept

Use of mHealth to provide targeted maternal or perinatal health information to women and their families. mHealth was defined as a medical and public health practice supported by mobile phones or tablets, making use of text, audio, images, video, or coded data in the form of voice calls, short messaging services (SMS), voice SMS, interactive voice response systems (IVRS), social media, mobile applications, third, and fourth generation mobile telecommunications.

### 2.3 Context

Studies conducted with women and family members living in India.

### 2.4 Search strategy

To identify relevant sources, four databases were searched: PubMed, CINAHL, Global Health, and ACM digital library.

The search strategy was created with an experienced librarian and further refined by the authors, including key search terms such as “perinatal”, “mhealth”, “maternal”, and “India”, with MeSH terms where possible. The final search strategy for each database can be found in (Supplementary File S1).

The databases were searched for relevant articles up to 3rd April 2023, with no specified earliest date as the term “mHealth” was not widely used prior to 2000. Final searches were uploaded into EndNote and duplicates were removed. Citations were then uploaded to Rayaan (rayaan.com). Texts were first screened by abstract, those that met inclusion criteria went on to full-text screen. Both stages of abstract screening and full text screening were conducted by two reviewers (ZS and ST) based on inclusion criteria. In addition, the references of shortlisted full text were hand-searched to identify further relevant studies, and subsequently included in the citation screening. Any disputes between reviewers were resolved article by article, as needed, by a third author (JEH).

### 2.5 Inclusion and exclusion criteria

Studies were included if they: (1) were peer reviewed; (2) were available in English; (3) described an mHealth intervention where health advice was provided over a technological medium directly to participants and families, without healthcare worker support. We included studies describing both single intervention delivery, such as through communication by SMS or phone call only, or multimodal intervention delivery which consisted of a package of mHealth messaging strategies such as both phone calls and SMS. We included both one-way communication and two-way communication (synchronous or asynchronous) strategies. We also included published study protocols.

Any studies performed outside of India, and multi-country studies where the results for India could not be isolated were excluded. We excluded mHealth interventions where digital content was delivered to participants via front line health workers (FLHW/s) such as Accredited Social Health Activists (ASHA), or mHealth interventions without a clear health messaging element (e.g., digital surveys or GPS tracking of women). We also excluded studies where the mHealth intervention was part of a broader complex intervention, or where the outcome of the mHealth component was not a primary intervention being tested.

We excluded non-peer-reviewed reports of technologies, such as dissertations, reports, books, web pages, and media releases. We excluded systematic reviews and meta-analyses following extraction of any relevant included articles.

### 2.6 Data extraction and synthesis

A draft data extraction table was finalised by the research team, following which data extraction tables were independently populated by two reviewers (ZS and ST), discussed, and later combined after confirmation by a third (JEH). Data extracted from the final studies included article characteristics (study authors, title, year, location of intervention, study type, sample size), technology used, intended user, duration of mHealth intervention, reported outcomes and technology feasibility and acceptability.

We used the mHealth evidence reporting and assessment (mERA), a WHO-designed checklist, to standardise mHealth evidence reporting and support replication of an intervention (14). We recorded whether each paper covered each domain and reported on checklist items poorly described across the identified papers. Given this was a scoping review to assess quantity and domains of evidence, study quality was not assessed. Patients and/or the public were not involved in the design, conduct, reporting, or dissemination plans of this research.

## 3 Results

### 3.1 Identification of eligible studies for review

**Figure 1:**
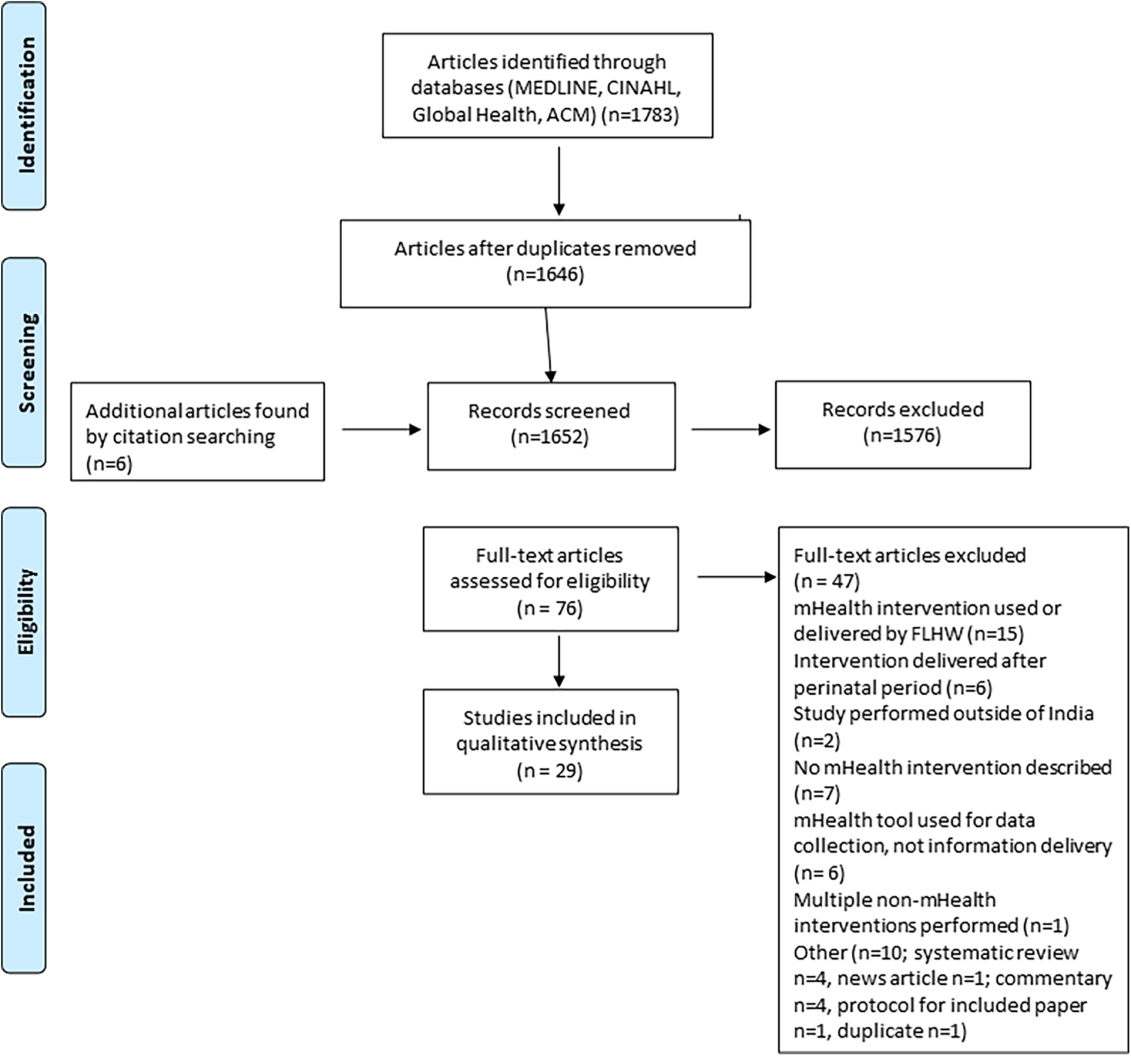
PRISMA flowchart for screening and selection of articles. FLHW, front line health worker.

The search process is outlined in Figure 1. The initial search was carried out on the 3rd of April 2023, yielding a total of 1,783 papers. 1,205 papers were identified from PubMed, 63 from CINAHL, 125 from Global Health, and 390 from ACM digital library. A total of 29 papers met the inclusion criteria, covering 22 different technologies. Three technologies were covered in more than one paper.

### 3.2 Scale and characteristics of included studies

Of the 29 papers identified, 11 were randomised control trials (RCTs) (15–25), eight were pre-post interventional studies (26–33) two were quasi-randomised control trials (34, 35), one was an interventional study without concurrent controls (36); seven papers were supplementary analyses of end user data or survey responses for already included papers (37–43). All studies identified are presented in Table 1, with further details provided in Supplementary File S2.

**Table 1:** Summary of 22 included mHealth technologies.

Study ID	Study type	Number of participants	Intervention summary
Yadav et al. (29)	Pre-post study	588	Technology: WhatsApp (social media) Description: clinician-moderated groups for women at different stages of pregnancy and the first year post-partum.
El Ayadi et al. (32) (MeSSSage)	Pre-post study (pilot)	29	Technology: Phone call, SMS, WhatsApp (social media) Description: Six weekly group calls for women in the first three months post-partum with SMS reminders and moderated WhatsApp groups throughout the intervention period.
Seshu et al. (27)	Pre-post study	12	Technology: IVRSDescription: Seven fortnightly audio dramas delivered to women screened positive for perinatal depression, educating on its prevention and management
Datta et al. (28)	Pre-post study (pilot)	120	Technology: SMS Description: Daily short messages (<90 characters) for 10 days on maternal and child health topics
Diamond-Smith et al. (26)	Pre-post study	3,455,950	Technology: Facebook (social media) Description: 6-week campaign of 84 adverts followed by a second 9-week campaign of 136 adverts on iron supplementation in women of childbearing age
LeFevre et al. (17, 37–39, 41, 42) (Kilkari)	RCT	>10,000,000	Technology: IVRS Description: Weekly stage-based, prerecorded calls on maternal and child health topics from 12 weeks’ gestation to one year postpartum
Johri et al. (23, 40) (Tika Vaani)	RCT (pilot)	185 (206 control)	Technology: IVRS Description: Weekly calls to primary caregivers of children aged 0–12 months with a health information capsule, reminder calls for infant vaccinations aligned with the national schedule and on demand access to IVRS content.
Basu et al. (19)	RCT (pilot)	38 (38 control)	Technology: SMS Description: 30 days of daily text messages to pregnant women <20 weeks’ gestation focused on oral hygiene and self-care practices
Manoharan et al. (20)	RCT	55 in mHealth study group (55 control group, 55 booklet group)	Technology: Phone call Description: Two reminder calls at 4 and 5 weeks’ post-partum to attend a 6-week appointment for blood glucose monitoring in women with gestational diabetes
Patel et al. (18)	RCT (pilot)	518 (518 control)	Technology: Phone call, SMS Description: Weekly phone counselling and daily reminder text messages on infant and young child feeding practices from the third trimester of pregnancy to 6 months’ post-partum
Murthy et al. (35) (mMitra)	Quasi-randomised control trial	1,516 (500 control)	Technology: IVRS Description: Twice weekly stage-based audio messages on maternal and child health topics from 6 weeks’ gestation to one year post-partum
Pawalia et al. (16)	RCT	12 (12 control group, 12 exercise only, 12 exercise and SMS advice)	Technology: SMS Description: Regular text messages providing dietary advice in pregnancy (frequency of messages not stated)
Irani et al. (33, 43) (Mobile Vaani)	Pre-post study	4,800	Technology: IVRS Description: Weekly outbound calls and on-demand access to content for mothers of children under 2 years old on maternal and child nutrition, family planning and management of diarrhoea
Yadav et al. (31) (Feedpal)	Pre-post study (pilot)	22	Technology: Chatbot (social media) Description: Three 10–30 min sessions with a chatbot that women 0–9 months’ post-partum could ask questions related to breastfeeding practices
Seth et al. (25)	RCT	201 SMS group (208 control, 203 SMS + compliance linked incentive)	Technology: SMS Description: reminder SMS message for each recommended immunisation for children aged 0–24 months according to the national schedule
Hazra et al. (34)	Quasi-RCT	640 in intervention group (authors compared responses of 428 from intervention area and 453 from control area)	Technology: voice messages Description: twice weekly voice messages to husbands of pregnant women on maternal and child health topics
Gupta et al. (36)	Pre-post study	Not stated	Technology: Phone call Description: Three counselling calls at 3–7, 20–42 and 42–60 days post-partum with family planning advice
Patel et al. (15) (M-SAKHI)	RCT (protocol)	N/A protocol	Technology: Phone call, voice message, SMS Description: Three SMS messages and one voice message per week, one phone to phone counselling session every 2 weeks on stage-based maternal and child health topics from 20 weeks’ gestation to one year post-partum. Additional alert SMS messages based on data entered by health workers (ASHAs)
Nayak et al. (22) (NeoRaksha)	RCT (protocol)	N/A protocol	Technology: SMS, app Description: SMS alerts to mothers of pre-term infants for immunisations according to the national schedule, alerts for hospital follow up visits, SMS messages with advice on care of pre-term infants and on-demand access to app content.
Sampathkumar et al. (30)	Pre-post study	682	Technology: SMS Description: Daily text messages for 100 days post-partum on maternal and child health topics
Bangal et al. (24)	RCT	200 intervention group (200 control group)	Technology: Voice message, SMS Description: voice message reminders to pregnant women of antenatal visits, educational SMS messages “at regular intervals” on maternal and child health topics
Pai et al. (21)	RCT	130 intervention group (130 control group)	Technology: voice message Description: three weekly messages to pregnant women for three months focused on antenatal nutrition and iron supplementation

This table outlines the basic characteristics of all included studies; a full version of this table with main findings can be found in Supplementary File S2.

Sample sizes varied between studies and could be divided into two categories: studies where participants were recruited or studies where participants could self-enrol. For the former category, sample sizes ranged from 12 (16, 27) to 1,516 participants (35). For studies which allowed participants to self-enrol, the recorded reach of intervention ranged from 44,664 (33) to 10 million participants (38).

We identified five studies where participants could self-enrol to the intervention. Kilkari is a large-scale intervention providing weekly IVRS audio calls from the second trimester of pregnancy to one year postpartum and has been scaled across 13 states, reaching over ten million subscribers by April 2019 (37). Other large-scale interventions included Mobile Vaani (33), clinician-moderated WhatsApp support groups (29) and a Facebook advertisement campaign addressing anaemia among women of reproductive age (26). Mobile Vaani is an IVRS providing participants with informative weekly outbound calls and on-demand access to content, that made 44,664 calls across February 2017 to July 2018. The moderated WhatsApp groups enabled participants to ask clinicians questions throughout the three trimesters of pregnancy and had involved over 3,000 members at time of writing.

Fifteen studies recruited participants from local hospitals or across defined sites. One example, MeSSSage, was trialled in the Mohali District of Punjab with 29 post-partum women (32). The six-week intervention combined weekly moderated group calls, on-demand access to educational audio recordings and a WhatsApp group, where participants shared stories, experiences, and asked questions. mMitra is a voice-based messaging service that delivered two messages per week from the sixth week of pregnancy to one-year post-partum (35). Women could not self-enrol, and they were recruited throughout pregnancy with controls matched by parity.

Two papers were study protocols, M-SAKHI and NeoRaksha. M-SAKHI comprises multiple components; those aimed at pregnant women included pushed text and voice messages, alert messages triggered by danger signs recorded in the app used by ASHAs, and fortnightly phone counselling (15). NeoRaksha is an intervention for mothers of preterm infants; mothers and ASHAs can enter data into the app, receive SMS alerts for immunisation and hospital appointment reminders, and information about caring for preterm infants at home (22).

### 3.3 Location

**Figure 2:**
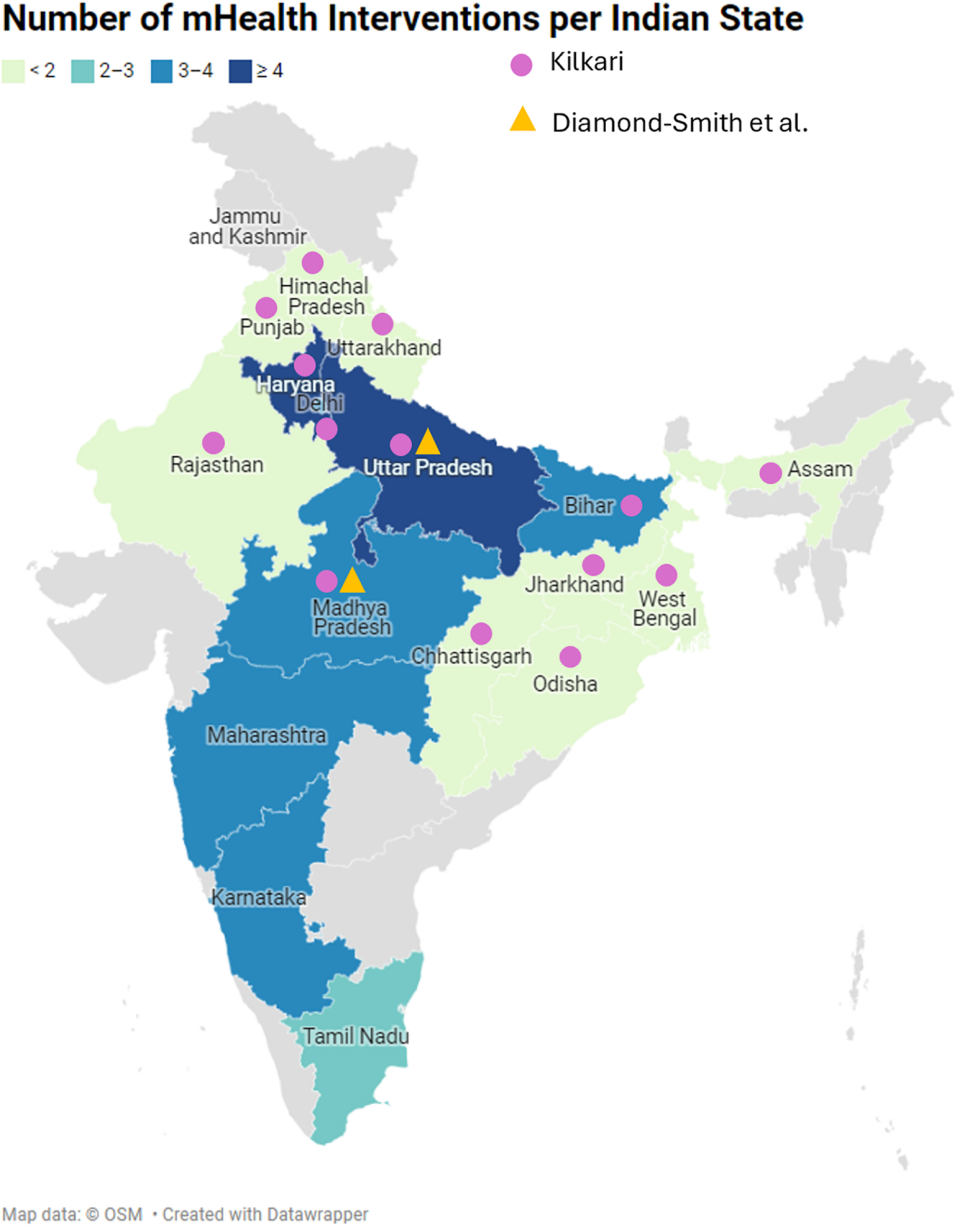
Distribution of mHealth interventions, of study interest, across India.

Most interventions were studied across a single state in India, with only two out of 22 interventions covering multiple states (Figure 2). Most interventions were performed in the Northen and Central zones of India, with the lowest number of interventions in the North Eastern zone, where the risks of maternal death are highest in rural and tribal areas (44). Kilkari, was piloted in Madhya Pradesh but has been scaled to 13 states (37), and in eight states was the only intervention active.

### 3.4 Recipient characteristics

Interventions varied slightly in their target audience. Four were targeted to pregnant women (16, 19, 21, 24), ten to those in the first-year post-partum (20, 22, 23, 25, 27, 30–33, 36) and six covered both pregnancy and post-partum periods (15, 17, 18, 29, 34, 35). Two studies targeted women planning for a child (26, 28). Most of the interventions were aimed at women, however, three interventions targeted women and their families (17, 28, 43) and one specifically targeted husbands (34).

To address low phone ownership amongst women in India, Hazra et al. (34) sent voice messages for four weeks to 640 husbands of pregnant women, covering a variety of maternal and neonatal health topics. Authors observed a significantly higher knowledge among listeners compared to non-listeners, including knowledge of one antenatal checkup in last trimester of pregnancy, receiving a postnatal checkup within seven days of delivery, and delayed bathing of newborn. However, only 34% of those who received the messages reported listening to them, and 53% of those did not discuss messages with their families because they considered themselves the family decision makers, highlighting traditional family customs impacting perinatal health knowledge and behaviours in India.

Diamond-Smith et al. (26) used Facebook adverts to inform of anaemia and the provision of iron and folic acid tablets to women planning for a child, reaching a total of 3,455,950 women across Madhya Pradesh and Uttar Pradesh. Authors measured the impact of the advert campaigns through pre- and post-intervention surveys. Outcomes of the campaign were mixed, however, two high focus indicators significantly improved after both campaigns: the odds ratio of a participant answering “yes” for “iron supplements can make labour/delivery more difficult,” was 0.790 compared to the baseline survey after the first campaign (*p* < 0.05), and 0.850 after the second (*p* < 0.01). For “iron supplements are only for women who have anaemia” the odds ratio of women answering “yes” was 0.885 after the first campaign compared to baseline (*p* < 0.05) and 0.897 after the second (*p* < 0.01). Whilst more studies are required, social media campaigns may be a useful approach to target women planning for pregnancy, who can be more difficult to reach once they have left school and are not usually attending regular hospital appointments.

### 3.5 Timing and frequency of messages

**Figure 3:**
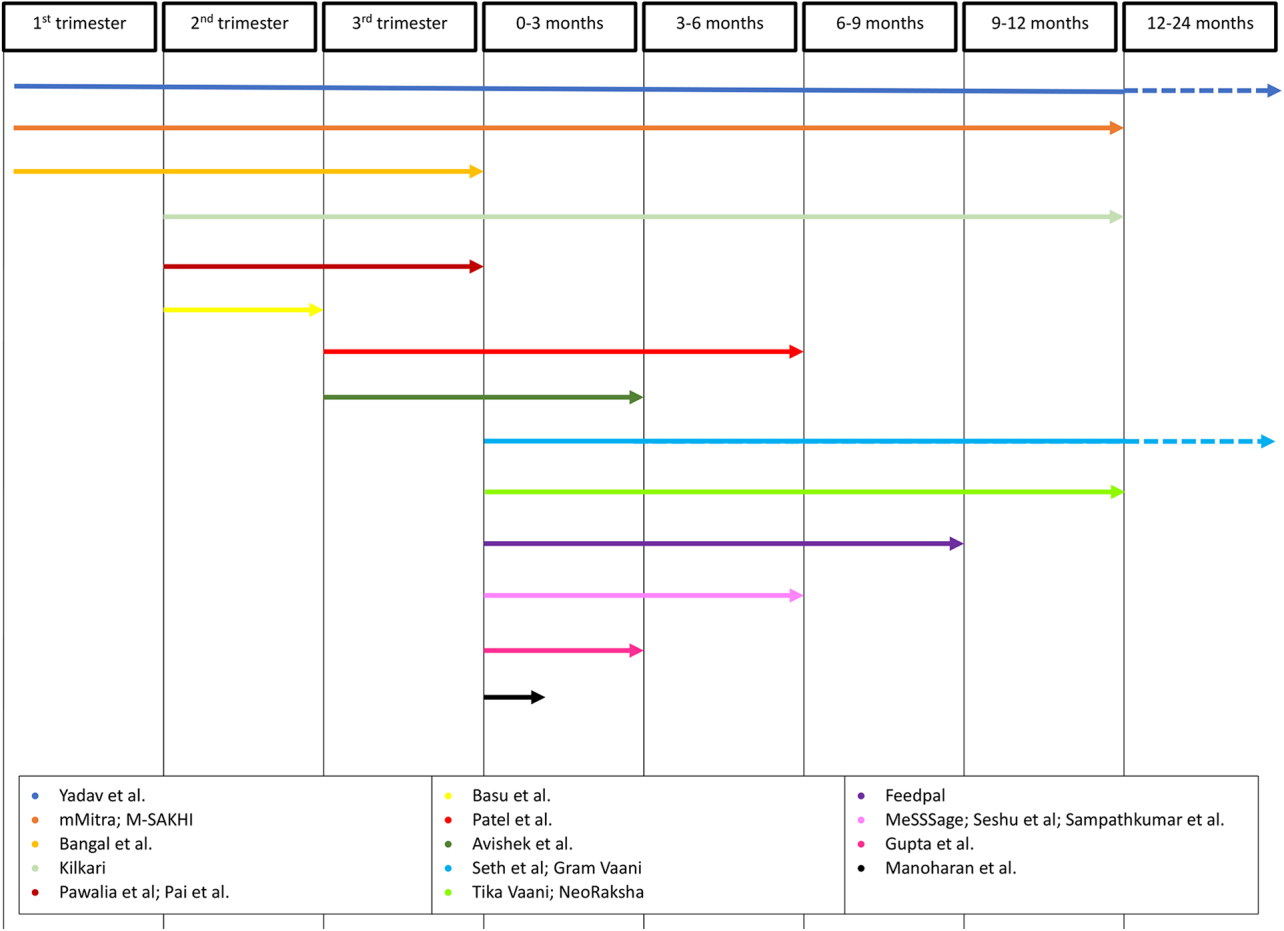
Timeline of mHealth interventions. Timeline arrows depict trimester of pregnancy and/or gestational age interventions covered.

Six interventions spanned the pregnancy and postpartum time periods (15, 17, 18, 29, 34, 35), whilst all other interventions targeted women planning for a child, pregnant, or post-partum only (Figure 3).

Three interventions allowed participant enrolment after the first stage of mHealth content delivery (29, 33, 37). Yadav et al. (29) studied the use of stage-based pre- and post-natal women and clinician WhatsApp groups; women could join the appropriate group for her stage at any time. However, staff-maintained records of participant details, such delivery date, were often incomplete as investigators had not adopted a consistent mechanism for demographic data collection at time of the study. This meant that moderators often had to contact a woman personally to request required data, a significant limitation to upscaling of the intervention. Kilkari allows activation of the service at any time from the second trimester of pregnancy until the child is aged one year, however, 58% of subscribers received and answered their first call after childbirth (37). The Kilkari profile database, MOTECH, is integrated with governmental databases that register pregnancies and births to match calls to pregnancy stage as accurately as possible. High listenership, defined as listening to at least 50% of the duration of one call, was similar across all the stages of the programme, suggesting that participants found the content useful whichever stage they joined at. Most other studies did not describe how they calculated gestational age.

Message frequency varied across interventions, ranging from daily or fortnightly contact to those timed to the national immunisation schedule or participant's antenatal or ASHA appointments. Most studies did not report the rationale for their chosen message frequency, but there appears to be an association with the mode of mHealth messaging. For instance, four interventions involved daily SMS messages (18, 19, 28, 30) whereas interventions involving phone calls or voice messages were more likely to be once or twice weekly (13, 15, 16, 19, 21, 30–33). This may be due to several factors, including the work required to produce the content and the risk of overloading a woman with too much information at once, leading to message fatigue and lack of engagement.

### 3.6 Type of technology and preferences

Most studies communicated to participants with one-way messaging modalities where researchers provided messages to women with no ability to send a response, with fewer studies enabling two-way messaging between participants and researchers (Table 2).

**Table 2:** Mode of mHealth technology for identified interventions.

	One-way communication	Two-way communication
Single intervention studies		
Social media (WhatsApp, Facebook)	1	2
SMS	5	0
IVRS	2	2
Voice messages	2	0
Phone calls	0	2
Multimodal intervention studies		
Information app + SMS alerts	0	1
IVRS, phone calls, and face to face meetings	0	1
SMS and voice calls/IVRS	1	3

Type of mHealth interventions identified, separated by one-way (investigator to participant) or two-way communication (investigator to participant, and participant to investigator), and by single and multimodal interventions.

#### 3.6.1 Short messaging service (SMS) and phone calls

Five studies used SMS to contact participants, three were RCTs, and two were feasibility studies. Datta et al. (28), sent text messages with perinatal health information to 120 families each day for 10 days, observing significant improvements across all maternal health, and over half of child health knowledge indicators tested. Three RCTs compared the impact of SMS reminders with control groups either having standard care, or a non-mHealth intervention. Seth et al. (25) sent 201 participants SMS reminders for each recommended DTP immunisation and found this did not significantly increase infant vaccination rates (40.1%) compared to normal care (41.7%). Basu et al. (19) provided 76 pregnant women with a one-time didactic education session; half of these participants then received a daily SMS reminder on oral hygiene for 30 days. Authors observed an improvement in twice-daily brushing frequency of the 38 participants who received the SMS compared to those who only received the education session, but this was not statistically significant (*P* = 0.43). Pawalia et al. (16) provided their pregnant participants with supervised exercise classes at the study hospital and regular text messages with dietary advice reminders. Whilst the SMS group lost more weight than the control group, a third study arm of participants who only attended the exercise classes lost the most weight. However, these results were not statistically significant.

Two studies contacted women through phone calls. Both were single-centre interventions targeting their patient population. Manoharan et al. (20) called 55 mothers with gestational diabetes mellitus (GDM) at fourth and fifth weeks post-delivery, observing no significant improvement in the number of participants who attended postnatal blood glucose monitoring. Interestingly, another arm of participants who received an informative leaflet did have a significantly higher attendance. Authors reasoned this was due to the visual aid and permanence of a leaflet reference material. Gupta et al. (36) observed an increase in post-partum family planning after a counsellor phoned postpartum women three times in a two-month period after delivery. However, these results were compared to the previous year's uptake, and their significance is not reported. Overall, SMS or voice call reminders did not significantly improve outcomes compared to control, or educational intervention arms.

#### 3.6.2 Voice messages and IVRS

Two studies contacted participants through voice messaging with varied outcomes (21, 34). Pai et al. (21) delivered voice messages to 130 participants to encourage iron supplementation and found no statistically significant improvement in haemoglobin level during follow up; Hazra et al. (34) found significant increases in knowledge levels for some but not all behavioural indicators in husbands who received voice messages.

Three IVRS interventions were evaluated with RCTs. IVRS enables users to interact with a telephone system using voice or keypad to play content (one-way communication), record messages or be connected to a clinician (two-way communication). mMitra evaluated 22 practice indicators, observing a significant improvement in tetanus toxoid injection, consulting a doctor if spotting/bleeding, saving money, and delivering in hospital (35). However, the control group performed significantly better than the intervention group receiving mMitra messages on two out of six practice indicators: resting regularly during pregnancy and home deliveries having a skilled birth attendant (35). Mobile Vaani observed that participants receiving their intervention had significantly higher knowledge than control participants for two of seven focus outcomes: knowledge of how to make child's food nutrient and energy dense and awareness of at least two modern spacing family planning methods, however, did not record if this translated into change in behaviour (33). Kilkari noted significant improvements in use of modern reversible contraceptives and immunisation of children at 10 weeks, but not for other maternal, newborn and child health outcomes assessed (17). The discrepancies in outcomes across IVRS studies is surprising and are likely due to a variety of reasons including self-reporting bias, attrition bias (all IVRS studies had high numbers lost to follow-up), contamination of control groups, and pre-existing participant preferences. For instance, the increase in contraceptive uptake observed in Kilkari is potentially skewed by the higher number of Kilkari participants with male children.

#### 3.6.3 Multimodal interventions

Six studies investigated the delivery of multimodal interventions (15, 18, 22–24, 32), two of these evaluated their intervention with randomised pilot studies (18, 23), and one through an RCT (24). In an RCT of 200 pregnant women recruited from a local hospital, Bangal et al. (24) observed that voice call reminders of antenatal appointments and educational SMS messages on perinatal health topics resulted in a statistically significant increase in antenatal visits, taking oral haematics, and higher post-natal checks ups in those receiving the intervention compared to those receiving normal care. Authors isolated these impacts to the phone call reminders which occurred 2 days before a woman's antenatal appointment, with participants reporting an increased feeling of trust and continuity of care by being reminded of, and therefore attending their appointments. Patel et al*.* (18) reported that the rate of timely initiation of breastfeeding was significantly higher in the 518 women who received once weekly mobile phone counselling from third trimester to 6 months postpartum and daily text messages on breastfeeding practices, compared to the 518 control women not receiving the intervention. However, intervention delivery was unblinded and randomised to hospital sites, so there may have been unidentified confounding variables between participants and differences in care provided between sites. Tika Vaani provided their vaccination intervention to 184 households with children aged between 0 and 12 months who resided in a study village. The Tika Vaani intervention consisted of educational audio capsules and voice reminders for immunizations broadcast via mobile phone and accessed through automated phone calls and free to dial IVRS. Face to face sessions were also provided to train women with the IVRS, aid community participation, and troubleshoot technology issues. Those receiving the Tika Vaani intervention had significantly higher basic health knowledge compared to the control group receiving usual services for 11 of 13 intermediate outcomes, such as knowing the immunisation schedule, however authors did not report if this translated into increased immunisation uptake (23).

### 3.7 Behaviours targeted

**Figure 4:**
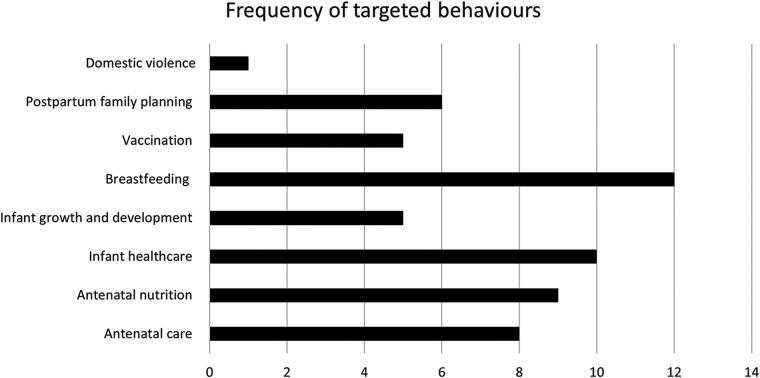
Frequency of targeted behaviours by mHealth interventions.

There was a wide variation in the scope of behaviours targeted by the mHealth interventions, with 14 studies targeting multiple behaviours and 8 targeting a single behaviour (Figure 4). Behaviours included iron supplementation for anaemia, postpartum glucose monitoring for those with gestational diabetes in pregnancy, antenatal care, breastfeeding, and postpartum family planning. Mobile Vaani authors found that diversifying their call content to include news, education and agriculture facilitated greater usage of the platform without coming at a cost to the listener rates of core content (43).

The most targeted behaviour across all studies was breastfeeding. Most papers advised exclusive breastfeeding up to six months of age (15, 18, 28, 29, 31, 33–35, 39), with some also advising mothers not to discard colostrum and to initiate breastfeeding within one hour of birth (34, 39). The rationale for targeting breastfeeding was consistent across studies, citing a high prevalence of malnutrition and stunting along with low rates of exclusive breastfeeding in India. Feedpal, a chat application where a researcher emulated the functionalities of a chatbot to answer participants queries, additionally cited the prevalence of traditional beliefs and practices deterring women from exclusive breastfeeding as a target for their intervention (31).

Three of five papers that evaluated an intervention for breastfeeding knowledge or practising of recommended techniques reported positive outcomes (18, 28, 34). However, two studies showed no significant difference in breastfeeding practices with an mHealth intervention, including the large Kilkari RCT (17, 33). Comparisons between different studies must be drawn cautiously, since they differed significantly in their intervention design, study design, target audience, follow up length, and outcome measures. For example, Patel et al. (18) was the only study where content focussed solely on breastfeeding, compared to a broader range of topic coverage. Their intervention was also high intensity, consisting of weekly phone counselling calls. Furthermore, mHealth messages were often interpreted within the context of cultural or personal beliefs. For instance, Kilkari advice to breastfeed exclusively was understood by some participants as advice to breastfeed primarily (39).

### 3.8 Evaluation of technology

mHealth technology was evaluated using a range of interviews, surveys, and engagement data. Eight out of 22 technologies did not provide any evaluation or plan to evaluate technology modality, seven of these studies were local or feasibility studies (16, 19–21, 24, 25, 36), and one was a protocol (22).

#### 3.8.1 System generated data

Five studies used collated system data, such as IVR channel skip rate, to determine user engagement with the technology (21, 26, 27, 34, 38). Kilkari reports that around 75% of all scheduled calls were answered and delivered, aided by an automated system that attempts to call subscribers up to nine times a week. Of answered calls, over 48% of calls were listened to for 50% of more of total content, and 43% were listened to for 75% of message duration, with most calls answered before 10am or after 6pm (37). Similarly, Mobile Vaani, tracks engagement to identify content that users skip, defined as listening to under 25% of content (43). Their platform also enables the recording of feedback by users, the number and content of which are analysed to provide insight on content that gains responses.

#### 3.8.2 Evaluative studies and feedback

We identified eight pre-post intervention studies, designed to evaluate the opinions of participants about the mHealth intervention (26, 27, 29–32, 35, 39). Of these, half were pilot studies (26, 27, 35, 38). All observed positive attitudes about the intervention. Of 19 interviewed MeSSSage participants, 84% were satisfied by the intervention, 100% of participants found the educational content useful, and 85% found the group discussions useful (32). Feedpal's authors found that 88% of 22 user's questions could be satisfactorily answered by their application, preferring shorter responses with images, with a few not reading longer, monotonous responses (31).

Eight technologies used, or planned to use, focus groups or in-depth interviews (22, 23, 25, 27, 29, 30, 34, 35), four used questionnaires (18, 29, 31, 43), and one used both to evaluate their technology (40). Seshu et al. (27) carried out in-depth interviews with 12 women with perinatal depression following their IVRS counselling intervention, finding that all women liked listening to the content. They found it improved their mood, and would continue the service, however, most participants expressed a preference for face-to-face contact over phone-based contact. Sampathkumar et al. (30) sent daily test messages over a 100-day period to mothers who delivered at their study site. Surveys and focus groups demonstrated that participants found receiving verified topical information made participants feel empowered in their infant's care, whilst the consistent messaging helped overcome anxiety and loneliness. However, some women found the stressful postnatal period meant they were distracted and unable to take in new information, and the main reasons for women who didn't activate the service were lack of understanding of the service or a lack of time to activate it.

#### 3.8.3 Barriers and facilitators to access

Limitations to women accessing mHealth interventions were commonly cited as household or childcare responsibilities (32, 33), lack of network coverage (20, 32, 39), or lack of physical access to phones (24, 36, 40, 41). Interventions consistently reported difficulty accessing women over the phone, who often share a phone with their husband who works throughout the day (27, 36, 40, 42). Four studies offered, or planned to offer, women phones to use for the duration of the study (15, 18, 31, 32). Nine studies rejected women without access to a phone (16, 17, 19, 21, 24, 25, 34, 35, 41), including Bangal et al. (24) where only 45% of eligible participants had access to a phone, therefore rejecting many of their target participants.

Where studies published the language of their messages, the majority were in Hindi, with few providing a selection of languages. Datta et al. (28) sent participants text messages in Tamil and English**,** finding 69.17% and 52.5% of respondents were “able to read” and “type and send” text messages, respectively, limited by high rates of illiteracy and absence of regional language font in mobile handsets. Similarly, Basu et al. identified a greater improvement in oral care in younger, more educated participants who received their text message intervention (19), and Mobile Vaani reported that older and less educated women found the intervention content, language, and dialect hard to understand and the pace of the storyline too fast (33). Tika Vaani found that high maternal education was predictive of higher uptake their mHealth audio vaccination reminders, whilst being a member of the lowest caste (social class) was associated with greater uptake of entertaining mHealth educational content, as this information was likely novel and useful (23).

#### 3.8.4 Infrastructure, interoperability, and data security

mERA checklist scoring revealed that network infrastructure, system interoperability, and data security protocols were poorly reported across studies. Only two studies included information on the network infrastructure required to support their mHealth intervention. Diamond-Smith et al. (26) justified using Facebook Ad Manager for their intervention due to the high rate of Facebook penetration amongst their target audience, quoting 2,900,000 monthly active users of their target demographic in Madhya Pradesh, and 6,500,000 users in Uttar Pradesh. Network connectivity was a limiting factor for participation in Kilkari, with women on the BSNL network excluded from participation (17).

Two studies reported on their data security protocols. Tika Vaani stored data on firewall protected servers (40). Feedpal required a Gmail account to login to the application, providing dummy accounts for those without Gmail accounts, and deleting all accounts and de-identifying patients prior to analysis (31). However, how and where this information was stored for analysis is not reported. Yadav et al. described multiple privacy breeches in their study of clinician moderated WhatsApp support groups, where male group members privately messaged women in the chat and shared jokes or religious or political propaganda (29). The platform moderator had limited control in countering these incidences by deleting these members, limited by the privacy functions of a pre-configured, and easily accessible, social media platform.

The interoperability between mHealth tool and health system was described by six interventions (15, 17, 22, 23, 26, 37–40, 43). NeoRaksha will enable participants to log infant development and health data into the android app that is viewable by healthcare professionals (22). M-SAKHI and Seth et al. provide supplementary technology to benefit ASHAs and public health workers alongside their direct to beneficiary mHealth interventions. M-SAKHI provides pushed text and voice messages to a participant when they deviate from recommended health practices, detection of which is enabled by health information logged by ASHAs on a corresponding android system (15). Seth et al. (25) couples an immunisation record keeping software with automated SMS reminders, proposing the identification system has further advantages for record keeping and tracking of missed or incomplete immunisations.

## 4 Discussion

To our knowledge, this is the only scoping review of mHealth interventions used by women and families for perinatal health promotion across India. We identified 29 studies describing 22 mHealth technologies. The most frequent behavioural targets for interventions were breastfeeding, antenatal nutrition, and infant healthcare. Most interventions communicated to women through one-way communication methods, most frequently through SMS. Reported barriers to the uptake of interventions included household or childcare responsibilities, technical difficulties, difficulty accessing a phone and difficulty understanding messages. Conversely, reported facilitators included group information sharing, use of additional non-maternal informative content, and a pictorial information format.

We found that single intervention studies sending participants one way communication reminders, such as SMS or phone calls, did not significantly improve antenatal visit attendance or other measured outcomes. This finding deviates from previous reviews which have concluded that mHealth reminders can improve attendance at maternal or antenatal appointments but have no consistent effects on perinatal health outcomes in low- and middle-income countries (45–47). Discrepancies may be due to differing follow up times across studies, the impact of a small number of studies, inclusion of complex interventions by other authors, as well as our inclusion of a variety of study types (46). Eleven of the 29 studies identified within the scope of our search were RCTs, and eight were pre-post-studies. The inclusion of studies other than RCTs may have led to discrepancies in our findings compared to previously published reviews. Particularly, the potential impact of pre-post studies included in our review must be acknowledged due to the potential for confounding variables such as, an increasing acceptance to mHealth through increased technology usage, and the impact of hormonal or family dynamic changes between data collection timepoints, which may exaggerate the impacts of mHealth interventions. Therefore, we emphasise the need for high quality RCTs investigating mHealth during the perinatal period, with long term follow up, to enable comparability across studies.

We have identified a limited number of studies utilising a multimodal delivery strategy and highlight the potential for multimodal mHealth interventions to improve perinatal health knowledge and outcomes. Multimodal intervention packages may offer visual elements, such as videos and photos, on-demand access to information, as well as reminders or counselling phone calls. These interventions may enhance behavioural change and increase appointment attendance, due to participants being able to refer to the material at later dates, share with family, or acknowledge information received in their preferred format (20, 31). Recent work performed outside the scope of our search recruited 286 anaemic pregnant women in Eastern India to receive a one-month intervention package of SMS reminders, audio messages, and phone calls (48). Authors observed a 44.9% increase in adherence to iron and folic acid tablets, compared to a 13.8% increase in the control group (*p* < 0.001). Providing women with a multimodal package of mHealth messaging alongside their pre-existing ASHA contact may provide an appropriate level of patient counselling whilst resolving staffing or economic gaps in resource-poor settings. However, multimodal interventions may bring further considerations such as risks of information overload and subsequent lack of adherence, which we identified as limitations reported by women even in single intervention studies (30). It will also be important to economically evaluate these interventions, to assess the cost-benefit of multiple interventions for service providers and for individual participants for whom multimodal interventions may cause extra costs such as travel to in person meetings, a need for a personal mobile phone, and needing to buy more airtime to call health professionals (49). Moreover, only one study from those we identified using multimodal interventions tried to isolate the impact of the individual components (24). Authors are therefore unable to extrapolate which arm of the intervention is driving any observed outcome. Future work on multimodal interventions should assess each modality individually as well as in combination to identify whether multimodal information delivery can lead to more effective behavioural change than single interventions. These studies must also assess the appropriate frequency, timings, and structure of delivered messages that lead to optimal intervention efficacy and participant engagement.

We have demonstrated that interventions targeting breastfeeding are capable of significantly increasing knowledge or practising of recommended breastfeeding techniques. These findings are in line with other reviews concluding that educational messaging can significantly improve the rate of exclusive breastfeeding in lower- and middle-income countries (9). However, participant's interpretation of breastfeeding messages is impacted by their traditional beliefs and understanding, limiting the generalisability of these results. Traditional feeding norms across India dictate that water, sweets, honey, and a ghutti are given alongside breastmilk, and pre-lacteal feeding is common with one study in Uttar Pradesh citing only 24% of women exclusively breastfeeding during the first 6 months (50). Work performed outside India has also demonstrated high early participant drop out of mHealth interventions targeting breastfeeding due to introduction of fluids during the neonatal period (51). We similarly observed evidence of cultural norms impacting the efficacy of contraceptive messaging. Therefore, effective mHealth studies should incorporate cultural norms and assess for appropriate understanding of messages when evaluating their interventions. To do so, an initial framework that enables study developers to understand specific factors that promote and discourage user adoption, including cultural norms, family preferences and traditional values will assist in targeting factors leading to early intervention failure.

This review highlights that that mHealth messages are an acceptable intervention to women and their families in the perinatal period. However, the inability to read and understand the messages was a common barrier against SMS and app-based interventions, consistent with findings in mHealth interventions across LMICs (52–54). Even studies that did not formally exclude illiterate participants are likely to be self-selecting due to trial advertising and motivation to take part. In India 83.0% of women aged 15–49 years old living in urban environments are literate compared to 65.9% in rural environments, where the risks of maternal death are highest (5). Written and inadequately translated messages may further widen the perinatal health gap between wealthy and poorer regions of the country, overall, limiting India in achieving the UN 2030 maternal and child mortality goals (44). Early work using large language models has shown potential for re-writing health messages into more accessible messaging suitable for lower literacy levels (55). However, further work must investigate whether this can be of use for accurate translation into local dialects and user-specific literacy levels. The potential advantages of these new technologies must overcome the limitations they may exacerbate such as the requirement for devices with advanced hardware and network connection, and the potential for widening the knowledge gap between participants of different literacy levels if messages are not adequately translated or technicalities are omitted.

We additionally highlight phone access as a limitation to scaling of mHealth interventions. Approximately half of women in Punjab share a phone with family members (56), and husbands can be “gatekeepers” to women's phone use (57); therefore, care must be taken when recruiting participants and designing interventions to ensure inclusion and avoid impacting household dynamics. A potential future direction is to encourage the active involvement of husbands in perinatal care interventions. A 2015 systematic review by Yargawa et al. evaluating 14 papers of male involvement in perinatal care in developing countries demonstrated this improved utilisation of maternal health and decreased the likelihood of antenatal depression by 90% (58). More rigorous studies are required to understand if husband and family involvement may enable wider access of women to mHealth interventions.

Furthermore, the lack of appropriate reporting of telecommunication network infrastructure, data security and interoperability as identified from the mERA checklist constitute novel findings from this review. These will be important for scalability, sustainability, and potential for long-term participant follow-up. An intervention's interoperability within the local healthcare system and the secure handling of participant data are both particularly important for studies working out of hospital sites or interventions utilising hospital records to ensure participants can make informed decisions about information sharing.

### 4.1 Strengths and limitations of our work

Strengths of our work include a rigorous methodology in line with JBI scoping review guidelines, developing a search strategy alongside an experienced librarian, and multiple reviewers assessing for inclusion or exclusion of papers. Limitations of our work include inclusion of only peer-reviewed published articles, thereby omitting potentially relevant data published in the grey literature. We did not review the health economic impacts of interventions, as this was outside the scope of our search, however, this will clearly be important when evaluating an intervention's long-term potential. We limited our search to studies published in English. Lastly, whilst we selected four research databases for our literature search to cover medical, allied health, global health, and computing fields- key themes of our work, these journals, and our limitation to English language, may not cover all relevant work that has been performed in India (59).

## 5 Conclusion

Overall, our review highlights the need for a framework to understand existing cultural beliefs and support structures, and to integrate these into mHealth technologies to avoid early intervention failure. Additionally, we highlight the need for rigorous RCTs investigating multimodal information delivery, and male involvement in mHealth interventions to support female participants. Standardisation of reporting of the development and implementation of mHealth interventions and monitoring and evaluation after deployment will be important for addressing the sustainability and efficacy of these interventions. The continual follow up of participants and interventions is necessary to demonstrate if there is lasting behavioural change, or repeated interest in mHealth interventions throughout subsequent pregnancies.

## Data Availability

The original contributions presented in the study are included in the article/Supplementary Material, further inquiries can be directed to the corresponding author.

## References

[1] Subramanian SV Ambade M Kumar A Chi H Joe W Rajpal S Progress on sustainable development goal indicators in 707 districts of India: a quantitative mid-line assessment using the national family health surveys, 2016 and 2021. Lancet Reg Health Southeast Asia. (2023) 13:7–8. 10.1016/j.lansea.2023.100155 PMC10306006 37383562

[2] United Nations Inter-agency Group for Child Mortality Estimation (UN IGME). Levels & Trends in Child Mortality 2020. New York: UNICEF (2020).

[3] The United Nations Maternal Mortality Estimation Inter-Agency Group (MMEIG). Trends in Maternal Mortality 2000 to 2020: Estimates by WHO, UNICEF, UNFPA, World Bank Group and UNDESA/Population Division. Geneva: World Health Organization (2023).

[4] Choorakuttil RM Rajalingam B Satarkar SR Sharma LK Gupta A Baghel A Reducing perinatal mortality in India: two-years results of the IRIA fetal radiology Samrakshan program. Indian J Radiol Imaging. (2022) 32(1):30. 10.1055/s-0041-1741087 35722649 PMC9200467

[5] International Institute for Population Sciences. National Family Health Survey (NFHS-4), 2015–16. Mumbai: DHS Program (2017).

[6] Singh S Rajak R. Autophagy-related biomarkers in preeclampsia: the underlying mechanism, correlation to the immune microenvironment and drug screening. BMC Pregnancy Childbirth. (2024) 24(1):1–14. 10.1186/s12884-023-06211-2 38166707 PMC10759589

[7] Sondaal SFV Browne JL Amoakoh-Coleman M Borgstein A Miltenburg AS Verwijs M Assessing the effect of mHealth interventions in improving maternal and neonatal care in low- and middle-income countries: a systematic review. PLoS One. (2016) 11(5):21–22. 10.1371/journal.pone.0154664 PMC4856298 27144393

[8] Director-General. mHealth use of Appropriate Digital Technologies for Public Health. Geneva: Seventy-First World Health Assembly (2018).

[9] Gayesa RT Ngai FW Xie YJ. The effects of mHealth interventions on improving institutional delivery and uptake of postnatal care services in low-and lower-middle-income countries: a systematic review and meta-analysis. BMC Health Serv Res. (2023) 23(1):1–16. 10.1186/s12913-023-09581-7 37296420 PMC10257264

[10] JBI Scoping Review Methodology Group. Scoping Reviews—Resources. Adelaide: JBI (2024). Available online at: https://jbi.global/scoping-review-network/resources (cited March 27, 2024).

[11] Tricco AC Lillie E Zarin W O’Brien KK Colquhoun H Levac D PRISMA extension for scoping reviews (PRISMA-ScR): checklist and explanation. Ann Intern Med. (2018) 169(7):467–73. 10.7326/M18-0850 30178033

[12] Peters MDJ Marnie C Tricco AC Pollock D Munn Z Alexander L Updated methodological guidance for the conduct of scoping reviews. JBI Evid Synth. (2020) 18(10):2119–26. 10.11124/JBIES-20-00167 33038124

[13] Guttmacher Institute. India. New York: Guttmacher Institute (2024). Available online at: https://www.guttmacher.org/regions/asia/india (cited March 27, 2024).

[14] Agarwal S Lefevre AE Lee J L’engle K Mehl G Sinha C Guidelines for reporting of health interventions using mobile phones: mobile health (mHealth) evidence reporting and assessment (mERA) checklist. Br Med J. (2016) 352:1–10. 10.1136/bmj.i1174 26988021

[15] Patel AB Kuhite PN Alam A Pusdekar Y Puranik A Khan SS M-SAKHI—mobile health solutions to help community providers promote maternal and infant nutrition and health using a community-based cluster randomized controlled trial in rural India: A study protocol. Matern Child Nutr. (2019) 15(4):1–16. 10.1111/mcn.12850 PMC6859979 31177631

[16] Alka P Sivachidambaram K Satya S Yadav VS. Exercise in pregnancy: effect on obesity parameters in Indian women—a randomized controlled trial. Rom J Diabetes Nutr Metab Dis. (2017) 24(4):315–23. 10.1515/rjdnmd-2017-0037

[17] LeFevre AE Shah N Scott K Chamberlain S Ummer O Bashingwa JJH The impact of a direct to beneficiary mobile communication program on reproductive and child health outcomes: a randomised controlled trial in India. BMJ Glob Health. (2022) 6(Suppl 5):e008838. 10.1136/bmjgh-2022-008838 35835477 PMC9288869

[18] Patel A Kuhite P Puranik A Khan SS Borkar J Dhande L. Effectiveness of weekly cell phone counselling calls and daily text messages to improve breastfeeding indicators. BMC Pediatr. (2018) 18(337):1–12. 10.1186/s12887-018-1308-3 30376823 PMC6206669

[19] Basu S Rajeev A Garg S Singh M. Effect of a text-messaging intervention on oral self-care practices in antenatal women in Delhi, India. Indian J Community Med. (2022) 47(1):133–7. 10.4103/ijcm.ijcm_929_21 35368494 PMC8971861

[20] Manoharan N Jayaseelan V Kar SS Jha N. Effectiveness of mobile call reminders and health information booklet to improve postnatal blood glucose monitoring among mothers with gestational diabetes Mellitus receiving care from a tertiary health centre, puducherry—a randomized controlled trial. Indian J Endocrinol Metab. (2022) 26(4):319–27. 10.4103/ijem.ijem_164_22 36185952 PMC9519841

[21] Pai N Supe P Kore S Nandanwar YS Hegde A Cutrell E Using automated voice calls to improve adherence to iron supplements during pregnancy: a pilot study. Proceedings of the Sixth International Conference on Information and Communication Technologies and Development: Full Papers—Volume 1 (2013). p. 153–63 10.1145/2516604.2516608

[22] Nayak BS Lewis LE Margaret B Bhat YR D’Almeida J Phagdol T. Randomized controlled trial on effectiveness of mHealth (mobile/smartphone) based preterm home care program on developmental outcomes of preterms: study protocol. J Adv Nurs. (2019) 75(2):452–60. 10.1111/jan.13879 30375032

[23] Johri M Chandra D Kone KG Sylvestre MP Mathur AK Harper S Social and behavior change communication interventions delivered face-to-face and by a mobile phone to strengthen vaccination uptake and improve child health in rural India: randomized pilot study. JMIR Mhealth Uhealth. (2020) 8(9):1–20. 10.2196/20356 PMC7546625 32955455

[24] Bangal VB Borawake SK Gavhane SP Aher KH. Use of mobile phone for improvement in maternal health: a randomized control trial. Int J Reprod Contracept Obstet Gynecol. (2017) 6(12):5458–63. 10.18203/2320-1770.ijrcog20175260

[25] Seth R Akinboyo I Chhabra A Qaiyum Y Shet A Gupte N Mobile phone incentives for childhood immunizations in rural India. Pediatrics. (2018) 141(4):1–9. 10.1542/peds.2017-3455 PMC5869335 29540571

[26] Diamond-Smith N Holton AE Francis S Bernard D. Addressing anemia among women in India-an informed intervention using Facebook ad manager. Mhealth. (2020) 6:39. 10.21037/mhealth-19-237a 33437835 PMC7793016

[27] Seshu U Khan HA Bhardwaj M Sangeetha C Aarthi G John S A qualitative study on the use of mobile-based intervention for perinatal depression among perinatal mothers in Rural Bihar, India. Int J Soc Psych. (2021) 67(5):467–71. 10.1177/0020764020966003 33059490

[28] Datta SS Pandiyan R Sivakumar KS. A study to assess the feasibility of text messaging service in delivering maternal and child healthcare messages in a rural area of Tamil Nadu, India. Australas Med J. (2014) 7(4):175–80. 10.4066/AMJ.2014.1916 24817911 PMC4009878

[29] Yadav D Dabas K Malik P Bhandari A Singh P. “Should I visit the clinic”: analyzing WhatsApp-mediated online health support for expectant and new mothers in rural India. Proceedings of the 2022 CHI Conference on Human Factors in Computing Systems (2022). 10.1145/3491102.3517575

[30] Sampathkumar S Sankar M Ramasamy S Sriram N Saravanan P Ram U. Uptake, engagement and acceptance, barriers and facilitators of a text messaging intervention for postnatal care of mother and child in India-a mixed methods feasibility study. Int J Environ Res Public Health. (2022) 19(15):1–12. 10.3390/ijerph19158914 PMC9329952 35897288

[31] Yadav D Malik P Dabas K Singh P. Feedpal. Proc ACM Human Comp Interact. (2019) 3:1–25. 10.1145/3359272

[32] Ayadi AM E Duggal M Bagga R Singh P Kumar V Ahuja A A mobile education and social support group intervention for improving postpartum health in Northern India: development and usability study. JMIR Form Res. (2022) 6(6):1–16. 10.2196/34087 PMC9280461 35767348

[33] Irani L Supriya V Ruchika M Verma RK Mohan D Dhar D Key learnings from an outcome and embedded process evaluation of a direct to beneficiary mobile health intervention among marginalised women in rural Bihar, India. BMJ Open. (2022) 12(10):1–14. 10.1136/bmjopen-2021-052336 PMC9558784 36207036

[34] Avishek H Khan ME Mondal SK. Mobile phone messaging to husbands to improve maternal and child health behavior in India. J Health Commun. (2018) 23(6):542–9. 10.1080/10810730.2018.1483444 29902122

[35] Murthy N Chandrasekharan S Prakash MP Ganju A Peter J Kaonga N Effects of an mHealth voice message service (mMitra) on maternal health knowledge and practices of low-income women in India: findings from a pseudo-randomized controlled trial. BMC Public Health. (2020) 20(1):1–10. 10.1186/s12889-020-08965-2 32487065 PMC7268375

[36] Nidhi G Akanksha G Sunita B Saroj S. Mobile technology for increasing postpartum family planning acceptability: the development of a mobile-based (mHealth) intervention through a dedicated counselor—a pilot innovative study conducted in a tertiary teaching hospital of Agra, Uttar Pradesh, India. J SAFOG. (2018) 10(1):74–80. 10.5005/jp-journals-10006-1564

[37] Bashingwa JJH Mohan D Chamberlain S Arora S Mendiratta J Rahul S Assessing exposure to kilkari: a big data analysis of a large maternal mobile messaging service across 13 states in India. BMJ Glob Health. (2021) 6(Suppl 5):4–5. 10.1136/bmjgh-2021-005213 PMC8327807 34312148

[38] Mohan D Bashingwa JJH Scott K Arora S Rahul S Mulder N Optimising the reach of mobile health messaging programmes: an analysis of system generated data for the kilkari programme across 13 states in India. BMJ Glob Health. (2022) 6(Suppl 5):1–6. 10.1136/bmjgh-2022-009395 PMC9366343 35940611

[39] Scott K Osama U Aashaka S Manjula S Shalini Y Anushree J Another voice in the crowd: the challenge of changing family planning and child feeding practices through mHealth messaging in rural central India. BMJ Glob Health. (2021) 6:1–15. 10.1136/bmjgh-2021-005868 PMC8327813 34312156

[40] Pérez MC Singh R Chandra D Ridde V Seth A Johri M. Development of an mHealth behavior change communication strategy: a case-study from rural Uttar Pradesh in India. COMPASS 2020—Proceedings of the 2020 3rd ACM SIGCAS Conference on Computing and Sustainable Societies (2020). p. 274–8

[41] Mohan D Scott K Shah N Bashingwa JJH Arpita C Osama U Can health information through mobile phones close the divide in health behaviours among the marginalised? An equity analysis of kilkari in Madhya Pradesh, India. BMJ Glob Health. (2021) 6:4–8. 10.1136/bmjgh-2021-005512 PMC8327823 34312154

[42] Chakraborty A Mohan D Scott K Sahore A Shah N Kumar N Does exposure to health information through mobile phones increase immunisation knowledge, completeness and timeliness in rural India? BMJ Glob Health. (2021) 6(Suppl 5):e005489. 10.1136/bmjgh-2021-005489 34312153 PMC8728358

[43] Chakraborty D Gupta A Seth A. Experiences from a mobile-based behaviour change campaign on maternal and child nutrition in rural India. Proceedings of the Tenth International Conference on Information and Communication Technologies and Development (2019). 10.1145/3287098.3287110

[44] Meh C Sharma A Ram U Fadel S Correa N Snelgrove JW Trends in maternal mortality in India over two decades in nationally representative surveys. BJOG. (2022) 129(4):550–61. 10.1111/1471-0528.16888 34455679 PMC9292773

[45] Mishra M Parida D Murmu J Singh D Rehman T Kshatri JS Effectiveness of mHealth interventions for monitoring antenatal care among pregnant women in low- and middle-income countries: a systematic review and meta-analysis. Healthcare (Basel). (2023) 11(19):4–14. 10.3390/healthcare11192635 PMC10572953 37830672

[46] Feroz A Perveen S Aftab W. Role of mHealth applications for improving antenatal and postnatal care in low and middle income countries: a systematic review. BMC Health Serv Res. (2017) 17(1):3–9. 10.1186/s12913-017-2664-7 29115992 PMC5678803

[47] Wagnew F Dessie G Alebel A Mulugeta H Belay YA Abajobir AA. Does short message service improve focused antenatal care visit and skilled birth attendance? A systematic review and meta-analysis of randomized clinical trials. Reprod Health. (2018) 15(1):191. 10.1186/s12978-018-0635-z 30466453 PMC6249748

[48] Sharma S Smitha MV Balakrishnan D. Telephonic intervention to combat non-adherence to oral iron-folic acid supplementation in pregnancy: a randomized controlled trial. Eur J Obstet Gynecol Reprod Biol X. (2023) 20:1–5. 10.1016/j.eurox.2023.100235 PMC10509657 37736306

[49] Musiimenta A Tumuhimbise W Pinkwart N Katusiime J Mugyenyi G Atukunda EC. A mobile phone-based multimedia intervention to support maternal health is acceptable and feasible among illiterate pregnant women in Uganda: qualitative findings from a pilot randomized controlled trial. Digit Health. (2021) 7:2055207620986296. 10.1177/2055207620986296 33717497 PMC7917428

[50] Population Council. Increasing Early and Exclusive Breastfeeding in UP: Implications for Behavior Change Communication, Policy Brief No. 5. New Delhi: Population Council (2010).

[51] Flax VL Negerie M Ibrahim AU Leatherman S Daza EJ Bentley ME. Integrating group counseling, cell phone messaging, and participant-generated songs and dramas into a microcredit program increases Nigerian women’s adherence to international breastfeeding recommendations. J Nutr. (2014) 144(7):1120–4. 10.3945/jn.113.190124 24812071 PMC4481538

[52] Kruse C Betancourt J Ortiz S Luna SMV Bamrah IK Segovia N. Barriers to the use of mobile health in improving health outcomes in developing countries: systematic review. J Med Internet Res. (2019) 21(10):1–13. 10.2196/13263 PMC6811771 31593543

[53] Peprah P Abalo EM Agyemang-Duah W Budu HI Appiah-Brempong E Morgan AK Lessening barriers to healthcare in rural Ghana: providers and users’ perspectives on the role of mHealth technology. A qualitative exploration. BMC Med Inform Decis Mak. (2020) 20(1):1–12. 10.1186/s12911-020-1040-4 32041608 PMC7011292

[54] Lund S Rasch V Hemed M Boas IM Said A Said K Mobile phone intervention reduces perinatal mortality in Zanzibar: secondary outcomes of a cluster randomized controlled trial. JMIR Mhealth Uhealth. (2014) 2(1):1–13. 10.2196/mhealth.2941 PMC4114456 25098184

[55] Loughran E Kane M Wyatt TH Kerley A Lowe S Li X. Using large language models to address health literacy in mHealth: case report. Comput Inform Nurs. (2024) 42(10):696–703. 10.1097/CIN.0000000000001152 38832874

[56] Pendse RS El Ayadi AM Sharma P Ahuja A Basavarajappa DH Duggal M Access to and use of mobile phone by postpartum, married women in Punjab, India: secondary analysis of mHealth intervention pilot data. JMIR Form Res. (2022) 6(5):1–8. 10.2196/34852 PMC9136645 35551059

[57] Hersh S Nair D Komaragiri PB Adlakha RK. Patchy signals: capturing women’s voices in mobile phone surveys of rural India. BMJ Glob Health. (2021) 6(Suppl 5):1–6. 10.1136/bmjgh-2021-005411 PMC8413869 34475116

[58] Yargawa J Leonardi-Bee J. Male involvement and maternal health outcomes: systematic review and meta-analysis. J Epidemiol Community Health (1978). (2015) 69(6):604–12. 10.1136/jech-2014-204784 PMC4453485 25700533

[59] Subramanyam N Krishnamurthy M Asundi AY. Indmed: an evaluative study on the coverage of Indian medical literature. J Inform Knowledge. (2017) 54:31–6. 10.17821/srels/2017/v54i1/101184

