# Supplementary material for: Mobile health (mHealth) interventions for health promotion during the perinatal period in India: a scoping review
Source: Front Glob Womens Health. 2024 Nov 27;5:1427285. doi: 10.3389/fgwh.2024.1427285 (PMC11631862; doi:10.3389/fgwh.2024.1427285)
Supplement: Supplementary file 1 [file Table1.docx]

**Supplementary file 1. mHealth literature search strategy**

**PubMed:**

((((pregnan*[Title/Abstract] OR gestation*[Title/Abstract] OR maternity*[Title/Abstract] OR perinatal*[Title/Abstract] OR postnatal*[Title/Abstract] OR maternal*[Title/abstract] OR prenatal*[Title/Abstract] OR antenatal*[Title/Abstract] OR post-natal*[Title/Abstract] OR pre-natal*[Title/Abstract] OR post-partum*[Title/Abstract] OR postpartum*[Title/Abstract]) OR ("Pregnancy"[MeSH Terms:noexp] OR "Pregnant Women"[MeSH Terms] OR "Maternal Health Services" OR "infant"[MESH])) AND ((((mHealth[Title/Abstract]) OR (Cell Phone[Mesh])) ) OR (("Computers, Handheld"[Mesh])))) OR (mobile*[Title/Abstract] OR smartphone*[Title/Abstract] OR iPhone*[Title/Abstract] OR iPad*[Title/Abstract] OR tablet computer*[Title/Abstract] OR cellphone*[Title/Abstract] OR SMS[Title/Abstract] OR ehealth*[Title/Abstract] OR e-health*[Title/Abstract] OR cell phone*[Title/Abstract] OR handheld computer*[Title/Abstract] or smartphone application*[Title/Abstract] or text messag*[Title/Abstract] or video messag*[Title/Abstract])) AND (India[MESH] OR Chandigarh[Title/Abstract] OR Delhi[Title/Abstract] OR Haryana[Title/Abstract] OR Himachal Pradesh[Title/Abstract] OR Jammu[Title/Abstract] OR Kashmir[Title/Abstract] OR Ladakh[Title/Abstract] OR Punjab[Title/Abstract] OR Rajasthan[Title/Abstract] OR Assam[Title/Abstract] OR Arunachal Pradesh[Title/Abstract] OR Manipur[Title/Abstract] OR Meghalaya[Title/Abstract] OR Mizoram[Title/Abstract] OR Nagaland[Title/Abstract] OR Tripura[Title/Abstract] OR Sikkim Chhattisgarh[Title/Abstract] OR Madhya Pradesh[Title/Abstract] OR Uttarakhand[Title/Abstract] OR Uttar Pradesh[Title/Abstract] OR Bihar[Title/Abstract] OR Jharkhand[Title/Abstract] OR Odisha[Title/Abstract] OR West Bengal[Title/Abstract] OR Dadra[Title/Abstract] OR Nagar Haveli[Title/Abstract] OR Daman[Title/Abstract] OR Diu[Title/Abstract] OR Goa[Title/Abstract] OR Gujarat[Title/Abstract] OR Maharashtra[Title/Abstract] OR Andhra Pradesh[Title/Abstract] OR Karnataka[Title/Abstract] OR Kerala[Title/Abstract] OR Puducherry[Title/Abstract] OR Tamil Nadu[Title/Abstract] OR Telangana[Title/Abstract])

**Global health:**

(pregnAN* or gestation* or maternity* or perinatal* or Infant* or postnatal* or maternal* or prenatal* or antenatal* or post-natal* or pre-natal* or post-partum* or postpartum*) AND (mHealth or computers, handheld or mobile* or smartphone* or iPhone* or iPad* or tablet computer* or cellphone* or SMS* or educational audio* or ehealth* or e-health* or cell phone* or handheld computer*or smartphone application or text messag* or video messag*) AND (India or (Chandigarh or Delhi or Haryana or Himachal Pradesh or Jammu or Kashmir or Ladakh or Punjab or Rajasthan or Assam or Arunachal Pradesh or Manipur or Meghalaya or Mizoram or Nagaland or Tripura or Sikkim Chhattisgarh or Madhya Pradesh or Uttarakhand or Uttar Pradesh or Bihar or Jharkhand or Odisha or West Bengal or Dadra or Nagar Haveli or Daman or Diu or Goa or Gujarat or Maharashtra or Andhra Pradesh or Karnataka or Kerala or Puducherry or Tamil Nadu or Telangana)).mp. [mp=abstract, title, original title, heading words, cabicodes words]

**CINAHL:**

((MH "Pregnancy") OR (MH "Pregnancy Trimester, First") OR (MH "Pregnancy Trimester, Second") OR (MH "Pregnancy Trimester, Third") OR (MH "Expectant Mothers") OR (MH "Perinatal Care") OR (MH "Postnatal Care+") OR (MH "Prenatal Care") OR (MH "Prepregnancy Care") OR TI ( gestation* or maternity* or perinatal* or postnatal* or infant* ) OR AB ( gestation* or maternity* or perinatal* or postnatal* or infant* )) AND ((MH "Telehealth") OR (MH "Cellular Phone") OR (MH "Text Messaging+") OR (MH "Smartphone") OR (MH "Computers, Hand-Held") OR (MH "Mobile Applications") OR TI ( mhealth or m-health or app or apps OR mobile* or smartphone* or iPhone* or iPad* or "tablet computer*" or cellphone* or SMS* or "voice messaging*" or "voice message*" or "educational audio*" or ehealth* or e-health* or "cell phone*" or "handheld computer*" ) OR AB ( mhealth or m-health or app or apps OR mobile* or smartphone* or iPhone* or iPad* or "tablet computer*" or cellphone* or SMS* or "voice messaging*" or "voice message*" or "educational audio*" or ehealth* or e-health* or "cell phone*" or "handheld computer*" )) AND ((MH "India") OR TI ( Chandigarh or Delhi or Haryana or Himachal Pradesh or Jammu or Kashmir or Ladakh or Punjab or Rajasthan or Assam or Arunachal Pradesh or Manipur or Meghalaya or Mizoram or Nagaland or Tripura or Sikkim Chhattisgarh or Madhya Pradesh or Uttarakhand or Uttar Pradesh or Bihar or Jharkhand or Odisha or West Bengal or Dadra or Nagar Haveli or Daman or Diu or Goa or Gujarat or Maharashtra or Andhra Pradesh or Karnataka or Kerala or Puducherry or Tamil Nadu or Telangana ) OR AB ( Chandigarh or Delhi or Haryana or Himachal Pradesh or Jammu or Kashmir or Ladakh or Punjab or Rajasthan or Assam or Arunachal Pradesh or Manipur or Meghalaya or Mizoram or Nagaland or Tripura or Sikkim Chhattisgarh or Madhya Pradesh or Uttarakhand or Uttar Pradesh or Bihar or Jharkhand or Odisha or West Bengal or Dadra or Nagar Haveli or Daman or Diu or Goa or Gujarat or Maharashtra or Andhra Pradesh or Karnataka or Kerala or Puducherry or Tamil Nadu or Telangana ))

**ACM:**

[All: pregnan* or maternal or perinatal* or postnatal*] AND [All: india*] AND [All: mhealth or mobile* or voice messaging*]
