# Supplementary material for: Mobile health (mHealth) interventions for health promotion during the perinatal period in India: a scoping review
Source: Front Glob Womens Health. 2024 Nov 27;5:1427285. doi: 10.3389/fgwh.2024.1427285 (PMC11631862; doi:10.3389/fgwh.2024.1427285)
Supplement: Supplementary file 2 [file Table2.docx]

**Supplementary file 2- Table of 22 included mHealth interventions**

| **Study** | **Study type** | **Technology** | **Number of participants reached** | **Intervention timing** | **Participant characteristics at time of intervention delivery** | **Behaviours targeted** | **Outcomes** |
| --- | --- | --- | --- | --- | --- | --- | --- |
| **Yadav et al.^29^** | Pre-post study | Social media (WhatsApp groups) | 588 | 8 groups:  - 3 trimesters of pregnancy  - 0–1-month infant  - 1-3 months  - 3-6 months  - 6-12 months  - 1-2 years | Pregnant / Post-partum | Antenatal care / breastfeeding / infant healthcare / infant development | Women used the group chats for consultation seeking 75% of the time, and information-seeking 25%. 50% of surveyed individuals stated they used the groups prior to going to a local clinic with health concerns. |
| **El Ayadi et al.^32^ (MeSSSage)** | Pre-post (pilot) study | Multimodal intervention: Group calls, SMS reminders and group chats | 29 | Weekly group calls for 6 weeks (infants aged 3-4 weeks at first call, all women had given birth within the last 3 months) with SMS reminder one day before, moderated SMS group chats during intervention period | Post-partum | Postpartum care/infant healthcare/infant development/breastfeeding/PPFP | 48% of participants attended ≥3 of the 6 calls; the main barriers were childcare and household responsibilities and network or phone issues. 84% of participants were very satisfied with the intervention; 100% found educational content, and 85% found group discussions very useful. |
| **Seshu et al.^27^** | Pre-post study | IVRS | 12 | Seven episodes released fortnightly - timing not stated | Pregnant/post-partum - screened positive for perinatal depression | Antenatal care / postpartum care / PPFP | 4 of 12 women interviewed had accessed the intervention in the preceding month, and most found it helpful. All women liked listening to the content on the IVRS, found it improved their mood, and would continue the service, however, the majority preferred face to face contact to phone-based contact. The intervention made one woman report it made her feel “lighter”. |
| **Datta et al.^28^** | Pre-post (pilot) study | SMS | 120 | Ten messages - one per day | Family members (62 male, 58 female participants) | Antenatal care / antenatal nutrition (iron supps) / infant healthcare / breastfeeding / vaccination | After receiving text messages, 67% of participants knew the minimum number of iron folic acid tablets to be consumed by a pregnant mother, as compared to 21.67% before receiving the text messages (P < 0.05). 52% of individuals knew about low-birth-weight babies after receiving text messages, as compared to 20% individuals before receiving texts (P < 0.05) |
| **Diamond-Smith et al.^26^** | Pre-post study | Social media (Facebook advertising campaign) | 3,455,950 | 6-week campaign of 84 adverts then 9-week campaign of 136 adverts | Women aged 18-49 | Antenatal nutrition (iron supplementation) | Survey outcomes to assess the impact of ad campaigns were mixed across indicators and between surveys. Two of thirteen knowledge indicators that were of high focus changed in the desired direction after both ad campaigns (“iron can make labour/delivery difficult” and “iron supplements are only for women who have anaemia”) |
| **LeFevre et al.^17(37-39,41,42)^ (Kilkari)** | RCT | IVRS | >10,000,000 | Weekly calls from 12 weeks gestation to one year postpartum | Pregnant / Post-partum | Antenatal care / antenatal nutrition / postpartum care / infant healthcare / breastfeeding / vaccination / PPFP | Over half of subscribers answer their first call after childbirth compared to during pregnancy. The system handles upwards of 1.2million calls per day on average and 50% of calls are picked up on the first call attempt, 76% by the third and 99.5% by the ninth call attempt. Exposure to Kilkari content on immunisation was significantly associated with an increase in men's knowledge (but not women's) about child immunisation (OR: 1.23, 95% CI 1.02 to1.48) and an increase in the timeliness of the child receiving vaccination at birth (Probit coefficient: 0.08, 95% CI 0.08 to 0.24). |
| **Johri et al.^23,40^ (Tika Vaani)** | RCT (pilot) | IVRS | 185 (206 control) | Multimodal intervention: Weekly call with health information capsule, weekly reminder capsule, three vaccine reminder calls aligned with national schedule, on demand access to IVRS content. | Primary caregivers of children aged 0-12 months at baseline (families) | Vaccination / infant healthcare | 67% of households participated in the mHealth strategy, 63% of households accessed vaccine reminders and 60% educational audio capsules. For 11 out of 13 intermediate outcomes relating to infant vaccination and child health knowledge, regression results showed significantly higher knowledge among the intervention group. |
| **Basu et al.^19^** | RCT (pilot) | Text messages | 38 (38 control) | Daily text message for 30 days. Pregnant <20 weeks gestation at start of intervention | Pregnant | Antenatal care (oral hygiene) | The proportion of participants reporting twice‑daily brushing frequency habits was higher in the mHealth compared to the comparison arm, but this difference was not statistically significant (P = 0.43). The twice‑brushing frequency increased in both the study arms, only the mHealth arm revealed a statistically significant reduction in the incidence of missed twice‑daily brushing episodes in the previous 7 days (P = 0.016). Participants in the mHealth arm also reported an improvement in the perceived attitudes toward oral health care during pregnancy (P = 0.006). |
| **Manoharan et al.^20^** | RCT | Voice call | 55 in mHealth study group  (55 control group, 55 booklet group) | Two calls - one at 4 and one at 5 weeks postpartum | Post-partum | Postpartum care (glucose monitoring for T2DM) | Out of 55 mothers with GDM in each arm, 56.60% who received a booklet, 42.3% who received mobile reminders, and 24.07% in the standard care arm had undergone postnatal blood glucose monitoring at 8 weeks. Participants who received a health information booklet had a 2.21 times increased chance of undergoing postnatal blood glucose monitoring compared to standard care arm after adjusting for age, pre-pregnancy BMI, family history of DM and treatment history, and this association was statistically significant (aRR: 2.21, 95% CI [1.35– 3.64], P < 0.002). The difference was not significant in the mobile reminder arm (1.65 [0.96–2.86], P value 0.072). |
| **Patel et al.^18^** | RCT (pilot) | Multimodal: Voice call + SMS | 518 (518 control) | Cell phone counselling once a week from third trimester to 6 months postpartum + daily text messages | Pregnant / Post-partum | Breastfeeding | Rate of exclusive breastfeeding was similar between groups at 24 h after delivery, but significantly higher in the intervention at all subsequent visits (control vs. intervention: 24 h: 74% vs 74%, p = 1.0; 6 wk.: 81% vs 97%, 10 wk.: 78% vs 98%, 14 wk.:71% vs 96%, 6 mo: 49% vs 97%, p < 0.001 for the last 4 visits). Adjusting for covariates, women in intervention were more likely to exclusively breastfeed than those in the control (AOR [95% CI]: 6.3 [4.9–8.0]). |
| **Murthy et al.^35^ (mMitra)** | Quasi-randomised control trial | IVRS | 1516 (500 control) | 2x a week from 6 weeks gestation until 1 year postpartum apart from daily for one week postpartum | Pregnant / Post partum | Antenatal care / antenatal nutrition (iron supps) / postpartum care / breastfeeding / vaccination / PPFP | The intervention group performed significantly better than controls on four maternal health practice indicators: observing a significant improvement in tetanus toxoid injection (1.596 (1.053–2.4128), p=0.028), consulting a doctor if spotting/bleeding (1.715 (1.070–2.748), p=0.025), saving money (1.790 (1.375–2.329), p=0.0001), and delivering in hospital (2.543 (1.488–4.348) p=0.001). The control group performed significantly better than the intervention group on two practice indicators: knowledge of how to make child’s food nutrient and energy dense ~~(~~treatment-on-treated: 18.8% (95% CI0.4% to 37.2%, p<0.045)) and awareness of at least two modern spacing family planning methods (treatment-untreated: 17.6% (95% CI 4.7% to 30.5%, p<0.008)). |
| **Pawalia et al.^16^** | RCT | SMS | 12  (12 control group, and 12 exercise only group) | Not stated, women >16 weeks gestation at recruitment | Pregnant | Antenatal nutrition | Both the exercise groups (exercise only, and exercise with SMS reminders) showed similar improvement in terms of all the parameters as compared to control, but there were no differences between exercise groups. Mean waist circumference gain was 5.45cm in the control group, 4.53cm in the exercise group and 3.57cm in the exercise and diet group (p=0.031). |
| **Irani et al.^33,43^ (Mobile Vaani)** | Pre-post study | IVRS | 4800 | On demand information + weekly outbound | Post-partum (families also included but not target group) | Antenatal nutrition / infant healthcare / breastfeeding / PPFP | Women in the intervention arm had significantly higher knowledge than women in the comparison arm for two of seven focus outcomes: knowledge of how to make child’s food nutrient and energy dense (treatment-on-treated: 18.8% (95% CI 0.4% to 37.2%, p<0.045)) and awareness of at least two modern spacing family planning methods (treatment-on-treated: 17.6% (95% CI 4.7% to 30.5%, p<0.008)). |
| **Yadav et al.^31^ (Feedpal)** | Pre-post study (pilot) | Social media (Chatbot) | 22 | 3x 10–30-minute sessions, mothers all 0-9 months postpartum | Post-partum | Breastfeeding | The majority (88%) of mother's questions could be answered by a chatbot application, queries are embedded in existing belief systems, 26% of queries related to breastfeeding problems and 18% related to conditions hindering exclusive breastfeeding. Most women liked the bot once they gained confidence in its credibility, and they appreciated the ability to talk at any time and anywhere. |
| **Seth et al.^25^** | RCT | SMS +/-compliance linked incentive (Indian Rupper Rs30 or $0.5 US dollars of phone time) | 201 SMS group  (208 control, 203 SMS + compliance linked incentive) | Reminder message for each recommended immunisation in first 2 years according to national schedule | Caregivers of children aged 0-24 months | Vaccination | Median immunization coverage at enrollment was 33% in all groups and increased to 41.7% (interquartile range [IQR]: 23.1%–69.2%), 40.1% (IQR: 30.8%–69.2%), and 50.0% (IQR: 30.8%–76.9%) by the end of the study in the control group, the group with mobile phone reminders, and the compliance-linked incentives group, respectively. The administration of compliance-linked incentives was independently associated with improvement in immunization coverage and a modest increase in timeliness of immunizations |
| **Hazra et al.^34^** | Quasi-RCT | Voice messages | 640 in intervention group  (authors compared responses of 428 from intervention area and 453 from control area) | Twice per week for four months | Husbands of women pregnant at start of the study (6 months or more gestation) | Antenatal care / postpartum care / breastfeeding / infant healthcare | Out of 428 husbands to whom voice messages were sent and subsequently interviewed, 34% reported that they had heard the messages. Of these, 46% discussed at least one of the messages with their wife or other family members. The level of knowledge among husbands who heard the intervention messages  were significantly higher for three of the five selected behaviours compared to those who did not hear the messages or were not exposed to the intervention messages. These include one antenatal checkup in last trimester of pregnancy, receiving a postnatal checkup within 7 days of delivery, and delayed bathing of newborn |
| **Gupta et al.^36^** | Intervention (no controls) | Voice call | Not stated | First call 3-7 days post-delivery, second 20-42 days post-delivery, third 42-60 days post-delivery | Post-partum | PPFP | The effective acceptance rates of postpartum family planning increased from 4.9% in 2012 to 29.7% in 2015 at the tertiary centre where the study was conducted. |
| **Patel et al.^15^ (M-SAKHI)** | RCT (protocol) | Multimodal: Voice calls + SMS messages | N/A protocol | 3x SMS messages per week, 1x voice message per week, 1x phone to phone counselling session every 2 weeks. Enrolment (within 20 weeks gestation) to 1 year postpartum | Pregnant / Post-partum | Antenatal nutrition / breastfeeding / infant growth and development / infant healthcare | N/A- protocol |
| **Nayak et al.^22^ (NeoRaksha)** | RCT (protocol) | Multimodal: information app + SMS messages | N/A protocol | Alerts for infant immunisations according to national schedule, alerts for routine hospital follow up visits, SMS messages with advice on care of pre-term infants. Intervention from day of NICU discharge until 1 year postpartum | Post-partum (mothers of pre-term infants admitted to NICU) | Infant healthcare / infant growth and development / breastfeeding | N/A- protocol |
| **Sampathkumar et al.^30^** | Pre-post study | SMS | 682 | One message per day for 100 days from activation of service postpartum (NB not necessarily first 100 days PP) | Post-partum | Postpartum care / infant healthcare / infant growth and development / breastfeeding | 60% of those offered accessed the SMS service. Top reasons for activation were to understand post-natal changes, continuation of care and clarification of conflicting intervention. Over 90% of users read messages daily and 80% were happy with message frequency. 30% of non-users had technical issues trying to activate it ad 17% did not have time to activate. 15% did not think it would be useful. |
| **Bangal et al.^24^** | RCT | Multimodal: Voice calls + SMS messages | 200 intervention group (200 control group) | Voice call reminders of antenatal visits, SMS messages “at regular intervals” (frequency not stated) | Pregnant | Antenatal care / antenatal nutrition / postpartum care | The intervention group attended more antenatal visits than the controls (57.5% vs 23.5%, p<0.0001), a higher proportion took oral haematinics for >3 months (81% vs 69%, p<0.0001),, rates of home births were lower (1 vs 6% although no indication of significance) and rates of post-natal checkup attendance were higher (85% vs 21%, p<0.0001). However, most of both groups gave birth in the rural hospital (85 vs 78.5%), |
| **Pai et al.^21^** | RCT | Voice messages | 130 intervention group (130 control group) | Calls three times per week for 3 months. Start point any time in second trimester | Pregnant | Antenatal nutrition | At enrolment, women had an average Hb level of 9.6 g/dL (WHO cutoff for anaemia is 11 g/dL). In the treatment group, average Hb levels increased by 0.32 g/dL, but the increase is not statistically significant (t(38)=- 1.74, p=0.09). In the control group, average Hb levels decreased by 0.10 g/dL. The difference of differences between treatment and control groups shows a positive trend (0.43 g/dL on average), but it is not statistically significant (t(77)=-1.52, p=0.13). |
